# A Study of the Strategic Alliance for EMS Industry: The Application of a Hybrid DEA and GM (1, 1) Approach

**DOI:** 10.1155/2015/948793

**Published:** 2015-03-04

**Authors:** Chia Nan Wang, Nhu Ty Nguyen, Thanh Tuyen Tran, Bui Bich Huong

**Affiliations:** ^1^Department of Industrial Engineering and Management, National Kaohsiung University of Applied Sciences, 415 Jiangong, Sanmin District, Kaohsiung 807, Taiwan; ^2^International Relations Office, Lac Hong University, No. 10 Huynh Van Nghe Street, Bien Hoa, Dong Nai 71000, Vietnam

## Abstract

Choosing a partner is a critical factor for success in international strategic alliances, although criteria for partner selection vary between developed and transitional markets. This study aims to develop effective methods to assist enterprise to measure the firms' operation efficiency, find out the candidate priority under several different inputs and outputs, and forecast the values of those variables in the future. The methodologies are constructed by the concepts of Data Envelopment Analysis (DEA) and grey model (GM). Realistic data in four consecutive years (2009–2012) a total of 20 companies of the Electronic Manufacturing Service (EMS) industry that went public are completely collected. This paper tries to help target company—DMU1—to find the right alliance partners. By our proposed approach, the results show the priority in the recent years. The research study is hopefully of interest to managers who are in manufacturing industry in general and EMS enterprises in particular.

## 1. Introduction

The Electronic Manufacturing Service (EMS), also known as Contract Manufacturing, has become a hot ticket industry nowadays as the people's demand on using more advanced technological, electronic devices is significantly increasing. This segment has witnessed the strong development throughout the last ten years. Therefore, EMS is remaining an important role in the country's long term prosperity [1]. Specifically, “It creates skilled jobs and generates revenues for national treasuries in the form of exports and investment. It also has a strong beneficiary role in terms of its contribution to the physical infrastructure of an economy, and spill-over effects to other areas such as science, construction and logistics” according to Global Manufacturing report (2010).

Today, the mentioned market is dominated by many EMS manufacturers, but this study only selects 20 enterprises which play major roles in the industry and can represent the whole industry in stock market; among them Hon Hai Precision Industry Co. Ltd. is the first rank. Founded in Taiwan by its current CEO Terry Gou in 1974, up to now, Hon Hai has more than 1,200,000 employees. The products vary in type including connectors, cables, enclosures, wired/wireless communication products, optical products, and power supply modules. Hon Hai Precision Industry Co., Ltd. offers these products for use in the information technology, communications, automotive equipment, precision molding, automobile, and consumer electronics industries. Its key customers consist almost entirely of the well-known high-tech companies such as Apple, Cisco, Dell, Nokia, and Sony.

**Figure 1:**
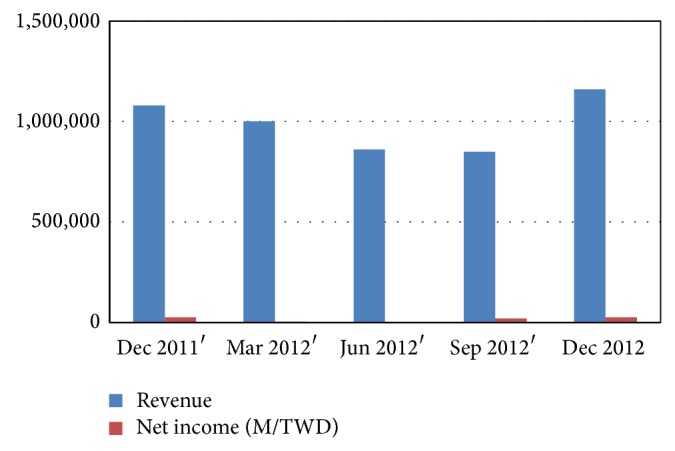
Revenue and net income of Hon Hai Precision Industry Co. Ltd. in period of 2011-2012.* (Source: Bloomberg News)*.

According to annual report of MMI, 2012, for the first time, this study found Top 50 sales reached a new high of $223.9 billion, evidencing an encouraging growth within the industry. Top 50 sales grew by 4.8% last year, despite end market weakness. However, without the contribution of industry giant Hon Hai Precision Industry, sales would have fallen by 5.0%. Hon Hai represented a 59% majority of Top 50 revenue in 2012.  Figure 1 demonstrates that the company is doing a great job which in turn helps strengthen the EMS sector.

The footprint of Hon Hai Precision Industry is all over the world in which China is the operation center; the rest of the plants are located in India, Malaysia, Japan, Brazil, Mexico, Hungary, Slovakia and Czech Republic, and Vietnam. For the future development planning, Foxconn Group expects to expand its production scale not only by looking for investment opportunity in more other territories but also by upgrading and building new factories in countries where it has already run its business.

The purpose of this research is to provide an assessment model based on Grey theory GM (1,1) and Data Envelopment Analysis (DEA) helping the target company—Foxconn or Hon Hai—to make a well-considered decision in finding the right partners. At the same time, the study also provides the prediction about firms' business in the future, which is relevant for them when setting strategies for production capacity planning and for investment decision making and whether they should expand their business in international market or not. The results of the case study can be the reference of the strategic alliances partner selection for worldwide EMS providers.

## 2. Literature Review

### 2.1. Strategic Alliances

Mockler [2] considers the agreements between companies (partners) to reach objectives of common interest. Strategic alliances are among the various options which companies can use to achieve their goals; they are based on cooperation between companies. This point of view was shared by Parkhe [3]: “Strategic alliance means the permanent cooperative agreement between the companies, including the inflow and linking of resources, and its cooperative purpose is finishing their company missions in strategic alliance.” These definitions emphasize the importance of the common business goals of the companies when carrying out alliance strategy.

Chan et al. [4] see strategic alliance as a cooperative agreement between various organizations. Its purpose aims at reaching the competitive advantage for enterprises that form a partnership. Strategic alliance brings together otherwise independent firms to share resources in product design, production, marketing, or distribution. In simple words, a strategic alliance is sometimes just referred to as “partnership” that offers businesses a chance to join forces for a mutually beneficial opportunity and sustained competitive advantage [5]. These definitions attach importance to competitive advantages among enterprises in strategic alliance.

Wang and Liu [6] researched the evaluation and selection of partner in logistics strategic alliance. The study establishes the index system of partners in logistics strategic alliance according to SCOR index model to evaluate the partners objectively. Based on AHP method, the researchers import the TOPSIS method to standardize the evaluation result so that more objective and effective evaluation result could be achieved and the appropriate partner is indicated finally.

Oh [7] focused on global strategic alliances in the telecommunications industry; the author points out the global marketplace is demanding an increasingly sophisticated, seamless worldwide communications network along with one inexpensive contract for every service. Global Strategic Alliances is a way of meeting these needs in the context of limited available resources.

Dittrich et al. [8] aimed to show that alliance networks can play an important role in facilitating large-scale strategic change projects. They focus on the particular case of IBM, whose radical redirection from an exploitation strategy towards an exploration strategy was realized by major changes in its network strategy. The findings of this paper suggest that the traditional view of large firms as being slow to adapt may not be valid because alliance networks can be used to overcome inertia.

### 2.2. Grey System Theory and DEA

Forecast is the explanation of something that has not yet been previously observed or is unknown. In recent years, many methods have been proposed for forecasting, especially forecasting in business such as fuzzy theory, neural networks, and grey prediction. Grey system theory, developed originally in early 1980s by Ju-Long [9], is an interdisciplinary scientific area. This theory has become a very popular method to deal with uncertainty problems under discrete data and incomplete information. Having superiority to conventional statistical models, grey models require only a limited amount of data to estimate the behavior of unknown systems [10].

Model Data Envelopment Analysis (DEA) was first described in a 1978 paper by Charnes, Copper, and Rhodes (CCR) [11]. In that work, the authors described a “data oriented” approach for evaluating the performance of a set of peer entities called Decision Making Units (DMUs) which convert multiple inputs into multiple outputs. The DMU can be banks, managers, shipping companies, or what we will evaluate in this paper, areas within EMS industry. Recent years had a great variety of application of DEA in both public and private sectors of different countries.

Chandraprakaikul and Suebpongsakorn [12] used data envelopment analysis to explore the operation performances of 55 Thai logistics companies from 2007 to 2010. The results point out the reasons for the inefficient Decision Making Units and provide improving directions for the inefficient companies accordingly.

Zhao et al. [13] made performance benchmarking by using Data Envelopment Analysis on Chinese coal mining industry. In the same time, they measured the changes of efficiency of coal mining industry by Malmquist Productivity Index.

A lot of methods are used for ranking and efficiency evaluation. However, most of the used models do provide no projection of Pareto efficiency. Thus, calculating the super efficiency becomes a significant issue [14]. Through this, Super SBM-I-V and grey model are integrated together to deal with the super efficiency and making strategy alliance partner.

Wang and Lee [15] focused on global strategic alliances in the hi-tech industries in Taiwan. By combining grey model and DEA, the researcher develops an effective method to help Taiwan's TFT-LCD industry to evaluate the operation efficiency and find the right candidate for alliances. The results of the study could assist companies' managers in making decisions in business extension.

## 3. Methodology

### 3.1. Research Process

**Figure 2:**
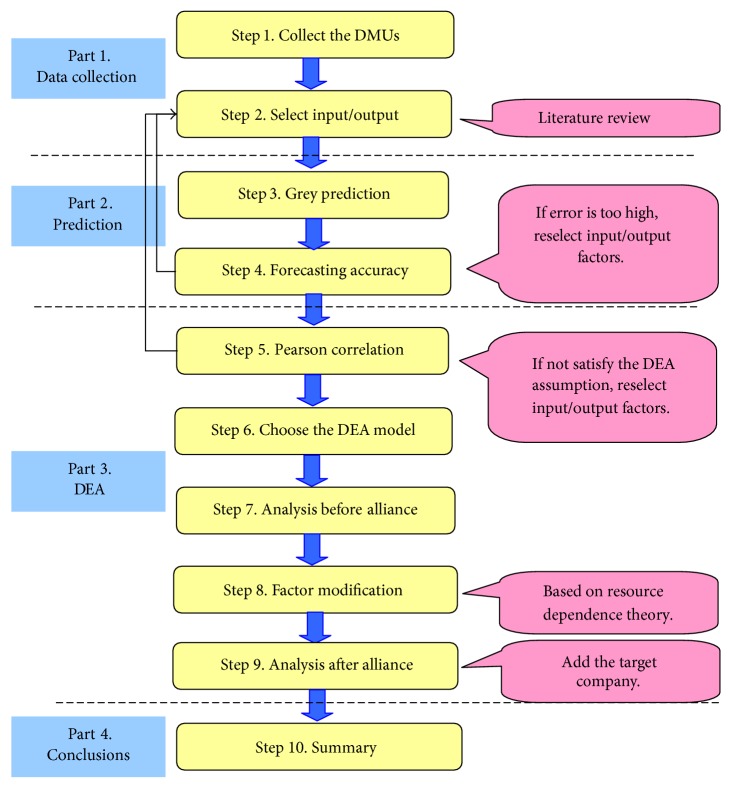
Research process.

Figure 2 shows details about this study step-by-step, and through this we can set up a whole image about how to integrate DEA and GM approaches. Step 1 and step 2 are about the setting stage mentioned earlier. In step 3 and step 4, grey prediction has been based on grey model GM (1,1) to predict the data values on 2013 and 2014. However, the forecast always show error. Therefore, in this study, the MAPE is applied to measure the forecasting error. If the forecasting error is too high, the study has to reselect the inputs and outputs.

In this paper, the software of DEA-Solver is employed to calculate super-SBM-I-V model. During step 5, the efficiency measuring by ranking DMUs' performance is then achieved. The formulation of DEA is to measure the efficiency of each decision making unit by constructing a relative efficiency score via the transformation of the multiple inputs and outputs into a ratio of a single virtual output to a single virtual input. Therefore, to test whether the data match with the basic assumptions of DEA methodology or not, correlation analysis of variables is calculated to verify for positive relationship between the selected inputs and outputs (step 6). If the variables get with the negative coefficient, they need to be removed, then we will go back to step 2 of the selection process to redo the variable selection until they can satisfy our condition. In this study, we employ the Pearson Correlation Coefficient Test.

The purpose of step 7 is to rank the efficiency of each decision making unit by applying the super-SBM-I-V model in the realistic data in 2012. In particular, by this way the researcher also can find out the target company's position in comparison with the other 19 EMS competitors. By step 8, a lot of information after analysis is given out since we have the results of step 7. However, the study does not recommend strategic alliance in this step until further analysis of step 9. In this step, the researcher has to stand on the side of the candidate companies which are selected for the target company's alliance to find the possible way of cooperation.

### 3.2. Collecting DMUs

After doing the survey the Electronic manufacturing services (EMS) market segments, the study finds out 20 enterprises in the MMI Top 50 list of the world's largest EMS providers. Then, the analysis was only conducted on the 20 companies which are stable in market and can provide the complete data for 4 consecutive years (2009–2012) in Bloomberg Business week news. Moreover, these 20 qualified companies play major roles in the EMS industry and can represent for whole industry in stock market (Table 1).

In this study, DMU1 is set as the target company with the headquarters located in Taipei, Taiwan. In the globalization and competition environment, strategic alliance could be a great way for DMU1 to require resources and extend its business map.

### 3.3. Nonradial Super Efficiency Model (Super-SBM)

In the present study, a DEA model “slack-based measure of super-efficiency” (super SBM) was used. This model was developed on “slacks-based measure of efficiency” (SBM) introduced by Tone [16].

In this model with *n* DMUs with the input and output matrices *X* = (*x*_*ij*_) ∈ *R* ^*m*×*n*^ and *Y* = (*Y*_*ij*_) ∈ *R* ^*s*×*n*^, respectively. *λ* is a nonnegative vector in *R* ^*n*^. The vectors *S* ^−^ ∈ *R* ^*m*^ and *S* ^+^ ∈ *R* ^*s*^ indicate the input excess and output shortfall, respectively.

The model formulation provides a constant return to scale as follows [17]:

(1) $$\begin{array}{c} \min\;\rho =\frac{1-\left(1/m\right)\sum_{i=1}^{m}s_{i}^{-}/x_{i0}}{1+\left(1/s\right)\sum_{i=1}^{s}s_{i}^{-}/y_{i0}} \end{array}$$

subject to

(2) $$\begin{array}{c} x_{0}=X\lambda +s^{-},\\ y_{0}=Y\lambda -s^{+},\\ \lambda \geq 0,\;s^{-}\geq 0,\;s^{+}\geq 0. \end{array}$$

The variables *S* ^+^ and *S* ^−^ measure the distance of inputs *Xλ* and outptut *Yλ* of a virtual unit from those of the unit evaluated. The numerator and the denominator of the objective function of model (1) measure the average distance of inputs and outputs, respectively, from the efficiency threshold.

Let an optimal solution for SBM be (*p* ^*^, *λ* ^*^, *s* ^−∗^, *s* ^+∗^). A DMU (*x*_0_, *y*_0_) is SBM-efficient if *p* ^*^ = 1. This condition is equivalent to *S* ^−∗^ = 0 and *S* ^+∗^ = 0, no input excesses and no output shortfalls in any optimal solution. SBM is nonradial and deals with input/output slacks directly. The SBM returns and efficiency measures between 0 and 1.

The best performers have the full efficient status denoted by unity. The super SBM model is based on the SBM model. Tone [16] discriminated these efficient DMUs and ranked the efficient DMUs by super-SBM model. Assuming that the DMU (*x*_0_, *y*_0_) is SBM-efficient, *p* ^*^ = 1, super-SBM model is as follows:

(3) $$\begin{array}{c} \min\;\delta =\frac{\left(1/m\right)\sum_{i=1}^{m}{\bar{x}}_{i}/x_{i0}}{\left(1/s\right)\sum_{r=1}^{s}{\bar{y}}_{r}/y_{r0}}, \end{array}$$

subject to

(4) $$\begin{array}{c} \bar{x}\geq \overset{n}{\underset{j=1,j\neq 0}{\sum }}\lambda_{j}x_{j},\\ \bar{y}\leq \overset{n}{\underset{j=1,j\neq 0}{\sum }}\lambda_{j}x_{j},\\ \bar{y}\geq x_{0},\;\bar{y}\leq y_{0},\;\bar{y}\bar{y}\geq y_{0},\;\lambda \geq 0. \end{array}$$

The input-oriented super SBM model is derived from model (3) with the denominator set to 1. The super SBM model returns a value of the objective function which is greater than or equal to one. The higher the value is, the more efficient the unit is.

As in many DEA models, it is crucial to consider how to deal with negative outputs in the evaluation of efficiency in SBM models too. However, negative data should have their duly role in measuring efficiency; hence, a new scheme was introduced in DEA-Solver pro 4.1 Manuel and the scheme was changed as follows.

Let us suppose *y*_*r*0_ ≤ 0. It has defined ${\bar{y}}_{r}^{+}$ and *y*_−*r*_^+^ by

(5) $$\begin{array}{c} {\bar{y}}_{r}^{+}=\underset{j=1,\ldots ,n}{\max}\left\{y_{rj}\;|\;y_{rj}>0\right\},\\ {\bar{y}}_{r}^{+}=\underset{j=1,\ldots ,n}{\min}\left\{y_{rj}\;|\;y_{rj}>0\right\}. \end{array}$$

If the output *r* has no positive elements, then it is defined as ${\bar{y}}_{r}^{+}=y_{-r}^{+}=1$. The term is replaced by *s*_*r*_^+^/*y*_*r*0_ in the objective function in the following way. The value *y*_*r*0_ is never changed in the constraints.

(1) ${\bar{y}}_{r}^{+}=y_{-r}^{+}=1$, the term is replaced by
  (6) $$\begin{array}{c} \frac{s_{r}^{+}}{y_{-r}^{+}\left({\bar{y}}_{r}^{+}-y_{-r}^{+}\right)/\left({\bar{y}}_{r}^{+}-y_{r0}\right)}. \end{array}$$
(2) (7) $$\begin{array}{c} \frac{s_{r}^{+}}{{\left(y_{-r}^{+}\right)}^{2}/B\left({\bar{y}}_{r}^{+}-y_{r0}\right)}, \end{array}$$

where *B* is a large positive number (in DEA-Solver *B* = 100).

In any case, the denominator is positive and strictly less than *y*_−*r*_^+^. Furthermore, it is inversely proportion to the distance ${\bar{y}}_{r}^{+}-y_{r0}$. This scheme, therefore, concerns the magnitude of the nonpositive output positively. The score obtained is units invariant; that is, it is independent of the units of measurement used.

### 3.4. Grey Forecasting Model

Although it is not necessary to employ all the data from the original series to construct the GM (1,1), the potency of the series must be more than four. In addition, the data must be taken at equal intervals and in consecutive order without bypassing any data [17]. The GM (1,1) model constructing process is described as follows.

Denote the variable primitive series *X* ^(0)^ as formula:

(8) X(0)=X01,X02,…,X0n,n≥4,

where *X* ^(0)^ is a nonnegative sequence. *n* is the number of data observed.

Accumulating Generation Operator (AGO) is one of the most important characteristics of grey theory with the aim at eliminating the uncertainty of the primitive data and smoothing the randomness. The accumulated generating operation (AGO) formation of *X* ^(0)^ is defined as

(9) X(1)=X11,X12,…,X1n,n≥4,

where

(10) $$\begin{array}{c} X^{\left(1\right)}\left(1\right)=X^{\left(0\right)}\left(1\right),\\ X^{\left(1\right)}\left(k\right)=\overset{k}{\underset{i=1}{\sum }}X^{\left(0\right)}\left(i\right),\;k=1,2,3,\ldots ,n. \end{array}$$

The generated mean sequence *Z* ^(1)^ of *X* ^(1)^ is defined as

(11) $$\begin{array}{c} Z^{\left(1\right)}=\left(Z^{\left(1\right)}\left(1\right),Z^{\left(1\right)}\left(2\right),\ldots ,Z^{\left(1\right)}\left(n\right)\right), \end{array}$$

where *Z* ^(1)^(*k*) is the mean value of adjacent data; that is

(12) Z1k=12X1k+X1k−1,k=2,3,…,n.

From the AGO sequence *X* ^(1)^, a GM (1,1) model which corresponds to the first order different equation *X* ^1^(*k*) can be constructed as follows:

(13) $$\begin{array}{c} \frac{dX^{1}\left(k\right)}{dk}+aX^{\left(1\right)}\left(k\right)=b, \end{array}$$

where parameters *a* and *b* are called the developing coefficient and grey input, respectively.

In practice, parameters *a* and *b* are not calculated directly from (13). Hence, the solution of above equation can be obtained using the least squares method. That is,

(14) $$\begin{array}{c} {\hat{X}}^{\left(1\right)}\left(k+1\right)=\left[X^{\left(0\right)}\left(1\right)-\frac{b}{a}\right]e^{-ak}+\frac{b}{a}, \end{array}$$

where *X* ^(1)^(*k* + 1) denotes the prediction *X* at time point *k* + 1 and the coefficients [*a*, *b*]^*T*^ can be obtained by the Ordinary Least Squares (OLS) method:

(15) $$\begin{array}{c} {\left[a,b\right]}^{T}={\left(B^{T}B\right)}^{-1}B^{T}Y,\\ Y=\left[\begin{array}{c} x^{\left(0\right)}\left(2\right)\\ x^{\left(0\right)}\left(3\right)\\ \vdots \\ x^{\left(0\right)}\left(n\right) \end{array}\right],\;\;B=\left[\begin{array}{cc} -z^{\left(1\right)}\left(2\right) & 1\\ -z^{\left(1\right)}\left(3\right) & 1\\ \vdots & \vdots \\ -z^{\left(1\right)}\left(n\right) & 1 \end{array}\right], \end{array}$$

where *Y* is called data series, *B* is called data matrix, and [*a*, *b*]^*T*^ is called parameter series.

We obtained ${\hat{X}}^{\left(1\right)}$ from (14). Let ${\hat{X}}^{\left(0\right)}$ be the fitted and predicted series:

(16) X^0=X01,X^02,…,X^0n,where  X^01=X01.

Applying the inverse accumulated generation operation (IAGO), namely,

(17) $$\begin{array}{c} X^{\left(0\right)}\left(k+1\right)=\left[X^{\left(0\right)}\left(1\right)-\frac{b}{a}\right]e^{-ak}\left(1-e^{a}\right). \end{array}$$

The grey model prediction is a local curve fitting extrapolation scheme. At least four data sets are required by the predictor (14) to obtain a reasonably accurate prediction [18].

### 3.5. Establishing the Input and Output Variables

In order to apply DEA model, it is particularly vital that inputs and outputs considered for the study be specified. Besides, using appropriate inputs and outputs should be considered carefully so that conclusions drawn may not be misled. By investigating some DEA literature reviews mentioned previously and the elements of the operation for EMS, the researchers decided to choose three inputs factors directly contributing to the performance of the industry including fixed assets, operating expenses, and cost of goods sold. The research selected the revenues, operating income, retained earnings as output factors because they are the important indexes to measure the performance of enterprises both in current and future situation (Table 2).

The study also applied DEA-based testing the correlation between input and output factors correlation, which will clearly show whether those variables are suitable or not. The result is indicated clearly in the next section.

## 4. Empirical Results and Analysis 

### 4.1. Forecasting Results

The researchers use GM (1,1) model to predict the realistic input/output factors for the next two years 2013 and 2014. In the Table 3, the study takes company DMU1 as an example to understand how to compute in GM (1,1) model in period 2009–2012.

This research selects the* fixed assets* of DMU1 as example to explain for calculation procedure, other variables are calculated in the same way. The procedure is carried out step by step as follows.

First, the researchers use the GM (1,1) model for trying to forecast the variance of primitive series.

1st: create the primitive series:

(18) $$\begin{array}{c} X^{\left(0\right)}=\left(7\text{,}871.4;9\text{,}130.60;11\text{,}922.80;13\text{,}094.50\right). \end{array}$$

2nd: perform the accumulated generating operation (AGO):

(19) $$\begin{array}{c} X^{\left(1\right)}=\left(7\text{,}871.4;17\text{,}002;28\text{,}924.8;42\text{,}019.3\right),\\ x^{\left(1\right)}\left(1\right)=x^{\left(0\right)}\left(1\right)=7\text{,}871.4,\\ x^{\left(1\right)}\left(2\right)=x^{\left(0\right)}\left(1\right)+x^{\left(0\right)}\left(2\right)=17\text{,}002,\\ x^{\left(1\right)}\left(3\right)=x^{\left(0\right)}\left(1\right)+x^{\left(0\right)}\left(2\right)+x^{\left(0\right)}\left(3\right)=28\text{,}924.8,\\ x^{\left(1\right)}\left(4\right)=x^{\left(0\right)}\left(1\right)+x^{\left(0\right)}\left(2\right)+x^{\left(0\right)}\left(3\right)+x^{\left(0\right)}\left(4\right)=42\text{,}019.3. \end{array}$$

3rd: create the different equations of GM (1,1).

To find *X* ^(1)^ series and the following mean obtained by the mean equation is

(20) $$\begin{array}{c} z^{\left(1\right)}\left(2\right)=\frac{1}{2}\left(7\text{,}871.4+17\text{,}002\right)=12\text{,}436.7,\\ z^{\left(1\right)}\left(3\right)=\frac{1}{2}\left(17\text{,}002+28\text{,}924.8\right)=22\text{,}963.4,\\ z^{\left(1\right)}\left(4\right)=\frac{1}{2}\left(28\text{,}924.8+42\text{,}019.3\right)=35\text{,}472.05. \end{array}$$

4th: solve equations.

To find *a* and *b*, the primitive series values are substituted into the Grey differential equation to obtain

(21) $$\begin{array}{c} 9\text{,}130.60+a\times 12\text{,}436.7=b,\\ 11\text{,}922.80+a\times 22\text{,}963.4=b,\\ 13\text{,}095.50+a\times 35\text{,}472.05=b. \end{array}$$

Convert the linear equations into the form of a matrix.

Let

(22) $$\begin{array}{c} B=\left[\begin{array}{cc} -12\text{,}436.7 & 1\\ -22\text{,}963.4 & 1\\ -35\text{,}472.05 & 1 \end{array}\right],\;\;\hat{\theta }=\left[\begin{array}{c} a\\ b \end{array}\right],\\ y_{N}=\left[\begin{array}{c} 9\text{,}130.60\\ 11\text{,}922.80\\ 13\text{,}095.50 \end{array}\right]. \end{array}$$

And then use the least squares method to find *a* and *b*:

(23) $$\begin{array}{c} \left[\begin{array}{c} a\\ b \end{array}\right]=\hat{\theta }={\left(B^{T}B\right)}^{-1}B^{T}y_{N}=\left[\begin{array}{c} -0.169642810\\ 7374.98 \end{array}\right]. \end{array}$$

Use the two coefficients *a* and *b* to generate the whitening equation of the differential equation:

(24) $$\begin{array}{c} \frac{dx^{\left(1\right)}}{dt}-0.169642810\times x^{\left(1\right)}=7374.98. \end{array}$$

Find the prediction model from equation

(25) X1k+1=X01−bae−ak+ba,x1k+1=7,871.4−7374.98−0.169642810e0.169642810×k+7374.98−0.169642810=51344.98·e0.169642810×k−43473.6.

Substitute different values of *k* into the equation

(26) k=0 X11=7871.4,k=1 X12=17364.14,k=2 X13=28611.92,k=3 X14=41939.20,k=4 X15=57730.45,k=5 X16=76441.21.

Derive the predicted value of the original series according to the accumulated generating operation and obtain

(27) x^(0)(1)=x(1)(1)=7871.4 for  the  year  2009x^02=x^12−x^11=9492.74    forecasted  for  2010x^03=x^1(3)−x^1(2)=11247.78      forecasted  for  2011x^04=x^14−x^13=13327.28        forecasted  for  2012x^05=x^15−x^14=15791.25      forecasted  for  2013x^06=x^16−x^15=18710.76     forecasted  for  2014.

In the same with above computation process, the study could get the forecasting result of all DMUs in 2013 and 2014; the detail numbers are shown in Table 4.

### 4.2. Forecasting Accuracy

It is undeniable that forecasting always remains some errors; they are essentially about predicting the future in uncompleted information. Thus, in this paper, the MAPE (Mean Absolute Percent Error) is employed to measure the accuracy of a method for constructing fitted time series values in statistics. MAPE is often used to measure forecasting accuracy. In the book of Stevenson [19], it stated out clearly that MAPE is the average absolute percent error which measures of accuracy in a fitted time series value in statistics, specifically trending:

(28) MAPE=1n∑Actual−ForecastActual×100;n  is  forecasting  number  of  steps.

The parameters of MAPE state out the forecasting ability as follows:

 MAPE < 10% “Excellent,”
 10% < MAPE < 20% “Good,”
 20% < MAPE < 50% “Reasonable,”
 MAPE > 50% “Poor.”

The results of MAPE are displayed as in Table 5.

The calculations of MAPE are almost smaller than 10%, especially the average MAPE of 20 DMUs reaches 5.822% (below 10% as well), it strongly confirms that the GM (1,1) model provides a highly accurate prediction.

### 4.3. Pearson Correlation

To apply DEA model, we have to make sure the relationship between input and output factors is isotonic, which means if the input quantity increase, the output quantity could not decrease under the same condition [20]. In this study, firstly, the researcher conducted a simple correlation test—Pearson correlation—to measure the degree of association between two variables [21]. Higher correlation coefficient means closer relation between two variables while lower correlation coefficient means that they are less correlated [22].

The interpretation of the correlation coefficient is explained in more detail as follows. The correlation coefficient is always between −1 and +1. The closer the correlation is to ±1, the closer to a perfect linear relationship. Its general meaning was shown in Table 6.

In the empirical study, the bellowing results in Tables 7, 8, 9, and 10 indicate that the correlation well complies with the prerequisite condition of the DEA model because their correlation coefficient shows strong positive associations. Therefore, these positive correlations also demonstrate very clearly the fact that the researcher's choice of input and output variables at the beginning is appropriate. Obviously, none of variables removal is necessary.

### 4.4. Analysis before Alliance

This study executes the software of Super-SBM-I-V for the realistic data of 2012 to calculate the DMUs' efficiency and get their ranking before alliances. The empirical results are shown in Table 11.

The result indicated that the DMU14 has the best efficiency (the first ranking with the score = 3.9649155). 14 other companies including the target DMU1 also have good operation efficiency—in the 15th row. This ranking proves conclusively again that it is necessary for the target company to conduct strategic alliance to improve its performance.

### 4.5. Analysis after Alliance

According to the above calculated result before alliance, the target company got the score equal to 1, interpreting that its business in 2012 was good. However, the target company only is the 15th out of 20 companies. Guided by the business philosophy of developing constantly, this company should boldly improve its production efficiency by the formation of the alliance.

To implement the empirical research, the study starts to form virtual alliance and then executes DEA calculation. By combining the DMU1 with the rest of DMUs, the research gets 39 virtual alliances totally.

Here, the software of* DEA-Solver Pro 5.0* built by Saitech Company is used to calculate* Super-SBM-I-V model* for 39 DMUs. Table 11 shows the score and ranking results of virtual alliance in 2013.

Depending on the results depicted above, the research can easily compare the efficient frontiers between DMUs and virtual alliances. The changing from original target DMU1 to virtual alliance will clearly indicate the difference. The difference can be split into two groups. Positive results in difference demonstrate the alliance is better than original DMUs.* The more the difference is, the more efficient the alliance gets*. In contrast, the negative result means the alliance is worse. Tables 13 and 14, respectively, present the concrete result of two groups.

DMUs' ranking rose after alliance demonstrates the target company can take advantages from alliance.  Table 12 shows that 10 companies including DMU19, DMU5, DMU6, DMU4, DMU14, DMU10, DMU2, DMU16, DMU13, and DMU9 have the good characteristic and necessarily match with candidates' desire in doing business. The virtual companies (DMU19 + DMU1; DMU5 + DMU1; DMU6 + DMU1; DMU4 + DMU1) have the highest opportunities to have the best efficiency in applying strategic alliance business model (score > 1). Thus, those 4 candidates will be highly appreciated in considering strategic alliance. In particular, DMU19 is the best potential candidate for strategic alliance because the difference is the biggest, which is 10 (see Table 13). Therefore, DMU19 is the first priority for this strategy. The second group includes the companies in the category of the* not-good* alliance partnership.

According to data in Table 14, we can see quite clearly that 09 companies (DMU20, DMU8, DMU18, DMU7, DMU17, DMU3, DMU11, DMU15, and DMU12) get worse after strategic alliances (the DMUs' ranking reduced dramatically). Those companies would not be our choice due to non-benefits for Target Company.

### 4.6. Partner Selection

In previous section, the study finds the good alliance partnership based on the position of the target company DMU1. In reality, we need to analyze the possibility of alliance partnership against the category of the Good Alliance Partnership (see Table 13). We take the DMUs' ranking before alliance and after alliance of the companies in the category of the Good Alliance Partnership into consideration on their position to find out which companies are willing to cooperate with the target company.

As seen clear from Table 15, nine companies would not be willing to cooperate with the target company. The ranking of these companies after alliance reduced in comparison with original ones. It means the performance of the companies including DMU19, DMU5, DMU6, DMU4, DMU14, DMU10, DMU2, DMU16, and DMU9 is already good; they do not need to make the alliance partnership with the DMU1.

Therefore, total 9 above companies will have less desire to form an alliance with the target company because cooperation might reduce their performance.

By reviewing Tables 11 and 13 and checking the performance before and after the formation of an alliance, those figures clearly highlight the combination between* DMU13* and the target DMU1. Before alliance, the efficiency of* DMU13* does not reach the DEA frontier; however, the ranking of* DMU13* is improved after alliance with DMU1. It means the alliance can exhibit the good scenario for productivity improvement not only for the DMU1 but also for the* DMU13*. In the other words, by implementing alliance, both DMU1 and* DMU13* might have the chance to manage their resource more effectively. Hence, this research strongly recommends* DMU13* to cooperate with the target company DMU1.

Bsed on the findings of case study, the researchers would like to put forward recommendations of the strategic alliance partner selection for the target company DMU1 in order to improve its operation efficiency. The recommendations are presented clearly to make readers satisfy the questions “why is it necessary to follow such recommendations.” The noticeable candidates for alliance strategy are the company* DMU19* (the best efficiency improvement for the target company) and the company* DMU13* (the efficiency improvement for both target DMU1 and partner DMU13).

## 5. Conclusions

The pace of development in EMS industry is already mature and getting more and more competitive, resulting in the higher and higher demand for electronic devices and revenue market has become a battleground. Considering of these issues, enterprises always try to enhance their competitiveness or expand their business scale. These missions are assigned to enterprises' managers. That is the reason why a decision making approach on strategic alliance of EMS industry based on Grey theory and DEA is mainly raised in this study. This research focuses on the relationship between strategic alliance and firms' performance of EMS industry by using GM (1,1) and DEA model. The most important purpose of this study is to help the target company find the right partners for strategic alliance.

Many scholars and experts have already examined the related subjects of strategic alliance. In fact, strategic alliance is employed in the era of globalization while the competitiveness in the market become fierce, it helps firms to reduce risks and creates the mode of penetration. But how the strategic alliance leads the firms to be successful is the big challenge.

Basing on the realistic data in the past time (period of 2009–2012), the GM (1,1) model was used to foresee what will happen in terms of specific factors: fixed assets, cost of goods sold, operating expenses, revenue, operating income, and retained earnings. Predicting these factors is necessary for the enterprises reduce risks and improve the ability of reacting against different situations immediately. However, absolute accuracy is very hard to reach in forecasting; therefore, the MAPE is applied to this studies which the average MAPE values is 5.822%. The MAPE in this study confirms that GM (1,1) provides reliable and acceptable results which would be valuable information for firm's management board.

In the aspect of DEA model, it is based on the resource-based theory. And then, the study uses the Super-SBM model to evaluate the real firms individually and measure the operation performance of virtual decision making units for strategic alliances. The proposed methodology can easily identify and compare the efficiency before and after alliances.

In this research, DMU1, one of the EMS companies is employed to test whether the strategic alliance benefits exists if DMU1 has alliances with other companies in the same industry and give the firms suggestions and the direction of improvement. In Section 4, the study finds out that the following companies: DMU19, DMU5, DMU6, DMU4, DMU14, DMU10, DMU2, DMU16, DMU13, and DMU9 are the good candidates for DMU1 to have strategic alliances; in which the DMU19, DMU5, DMU6, and DMU4 are strongly recommended. In addition, the research also indicates a possible ideal alliance partners for DMU1. That is DMU13. Strategic alliance is not only good for the DMU1 but also good for the DMU13 as well.

Among the 39 combinations, the study finds that some companies' efficiency improved; however, some of them decreased, which indicates that strategic alliances maybe cope with many risks. Firms which have the better efficiency may lead to poor efficiency when the partners of strategic alliances have failed. On the contrary, firms which originally have poor efficiency may also have the chance to increase their own efficiency once they have chosen the right strategic alliance partner. Therefore, we can summarize that strategic alliances not always can help firms increase their efficiency and performance. Blindly proceeding strategic alliances may cause the company lose their overall competitiveness. The crucial thing in implementing strategic alliance is that enterprise should consider different aspects to get benefits. Once strategic alliance is considered and treated carefully and properly, firm's operation efficiency is surely raised.

This is a new studying method in both academic research and practical applications by combining Grey theory and Super—SBM model. The proposed method of this research not only forecasts some important business factors for EMS industry, but also provides an accurate and appropriate evaluation of the industry at current situation. That could be useful information helping EMS enterprises' top managers to have effective decision making for business strategy (including alliance strategy) in the future. The result after strategic alliance provides a meaningful reference to help many other industries' manager in finding the future candidates of strategic alliance.

Based on the results of this study, the researchers conclude clearly alliances model, and methodology which we bring up can also apply to the other industries to evaluate the strategic alliances partners' selection, to enhance the overall competitiveness, and to avoid the wrong strategic alliances.

Although the paper shows that GM (1,1) is a very flexible and easy model of use to predict what would happen in the future of business and DEA is an efficient tool to measure the firms' performance, we still cannot deny some restrictions about these two methods for further studies. Because the information of some nonlisted companies is difficult to obtain, the study was conducted only with the data of 20 companies in Top 50 EMS providers. The selection of input and output variables seem not completely to reflect the overall EMS industry. Therefore, the limited number of DMU and input-output variables could leave the issues open for debate.

In actual alliances or union, the enterprises may have different considerations, such as the industry expansion, technology acquisition, and market development. As long as we can properly adjust the input and output factors through the method applied and the process established in this study, we can still get results with the reference value.

## Figures and Tables

**Table 1:** List of EMS companies.

Number order	Code DMUs	Companies	Headquarter addresses
1	DMU1	Hon Hai Precision Industry	New Taipei, Taiwan
2	DMU2	Jabil Circuit Inc.	St. Petersburg, FL
3	DMU3	Sanmina Corporation	San Jose, CA
4	DMU4	Shenzhen Kaifa Technology Co., Ltd.	Shenzhen, China
5	DMU5	Benchmark Electronics Inc.	Angleton, TX
6	DMU6	Plexus Corp.	Neenah, WI
7	DMU7	Kitron	Billingstad, Norway
8	DMU8	Di-Nikko Engineering Co., Ltd.	Nikko, Japan
9	DMU9	Fabrinet	Pathumthani, Thailand
10	DMU10	Flextronics Int'l Ltd.	Singapore
11	DMU11	Hana Microelectronics Pcl	Bangkok, Thailand
12	DMU12	Global Brand Manufacture Ltd.	New Taipei, Taiwan
13	DMU13	SIIX Corporation	Osaka, Japan
14	DMU14	Venture Corporation Limited	Singapore
15	DMU15	Orient Semiconductor Electronics Ltd.	Kaohsiung, Taiwan
16	DMU16	Universal Scientific Industrial (Shanghai) Co., Ltd.	Shanghai, China
17	DMU17	VS Industry Berhad	Senai, Malaysia
18	DMU18	Integrated Micro-Electronics, Inc.	Laguna, Philippines
19	DMU19	Celestica Inc.	Toronto, Canada
20	DMU20	PartnerTech AB	Vellinge, Sweden

Source: the MMI Top 50 for 2012.

**Table 2:** Inputs and outputs data of all DMUs in 2012.

DMUs	Inputs (by 1,000,000 U.S. dollars)			Outputs (by 1,000,000 U.S. dollars)		
	(I) Fixed assets	(I) Cost of goods sold	(I) Operating expenses	(O) Revenues	(O) Operating Income	(O) Retained earning
DMU1	13,094.5	119,144.5	7,420.5	130,088.7	3,613.6	14,826.1
DMU2	1,779.2	15,843.4	687.1	17,151.9	621.4	766.9
DMU3	569.4	5,657.6	265.8	6,093.3	170	−4,960.9
DMU4	225.5	2,607.4	60.2	2,676.6	7.2	378.8
DMU5	176.1	2,291.4	90	2,468.2	86.8	493.7
DMU6	265.2	2,086.8	115.8	2,306.7	104.2	596.9
DMU7	21.9	175.1	101	277.3	11.3	−20.7
DMU8	43	341.6	18.2	368.2	11.7	23.7
DMU9	97.9	502.8	23.5	564.7	38.4	184.9
DMU10	2,174.6	22,187.4	834.8	23,569.5	547.3	−5,302.7
DMU11	226.3	497.6	37.6	524.7	31.9	402.6
DMU12	283.1	1,141.3	65.4	1,212.4	6.8	82
DMU13	147.3	1,813.5	71	1,912.6	47	240.9
DMU14	112.1	1,466.5	323	1,874.7	102.8	1,105.2
DMU15	215.7	324.4	23.1	354.8	8.3	−116.4
DMU16	179.7	1,912.6	142.3	2,173.8	121.4	290.5
DMU17	157.2	325.2	26.3	392.6	20.5	47
DMU18	88.1	609.2	44	661.8	8.7	93.1
DMU19	337	6,068.8	263.6	6,507.2	174.8	−2,097
DMU20	30.2	325.5	14.4	341.8	1.9	11.2

Sources: Bloomberg News.

**Table 3:** Inputs and outputs factors of DMU1 in period of 2009–2012.

DMU1	Inputs (by 1,000,000 U.S. dollars)			Outputs (by 1,000,000 U.S. dollars)		
	(I) Fixed assets	(I) Cost of goods sold	(I) Operating expenses	(O) Revenues	(O) Operating Income	(O) Retained earning
2009	7,871.4	59,064.00	3,433.10	65,260.40	2,781.90	8,833.50
2010	9,130.60	91,730.10	5,302.80	99,836.90	2,870.40	10,488.10
2011	11,922.80	106,167.50	6,157.70	115,008.80	2,760.40	12,561.00
2012	13,094.50	119,144.50	7,420.50	130,088.70	3,613.60	14,826.10

Sources: Bloomberg news.

**Table 4:** Forecasted values of inputs and outputs of all DMUs in 2013 and 2014.

DMUs	Inputs (by 1,000,000 U.S. dollars)						Outputs (by 1,000,000 U.S. dollars)					
	(I) Fixed assets		(I) Cost of goods sold		(I) Operating expenses		(O) Revenues		(O) Operating Income		(O) Retained earning	
	2013	2014	2013	2014	2013	2014	2013	2014	2013	2014	2013	2014
DMU1	15,791.25	18,710.76	135,870.33	154,613.11	8,724.06	10,337.24	148,412.31	169,244.08	3,942.1	4,475.78	17,573.62	20,867.92
DMU2	1,976.27	2,184.79	18,168.59	20,389.86	701.28	725.14	19,683.74	22,105.41	821.54	1,029.47	1,513.14	3,035.01
DMU3	575.04	574.60	5,689.22	5,605.09	265.63	264.02	6,120.42	6,014.75	169.91	154.02	−4,860.51	−4,743.71
DMU4	223.32	217.90	2,32.82	2,064.27	70.44	84.68	2,388.01	2,126.07	6.41	3.79	382.52	383.78
DMU5	209.11	243.47	2,286.19	2,327.13	88.44	87.37	2,443.02	2,478.16	67.41	63.11	538.54	588.82
DMU6	280.57	297.71	2,260.20	2,422.78	121.21	125.83	2,489.36	2,660.29	108.27	112.51	693.02	799.09
DMU7	22.10	21.72	174.77	174.27	100.64	100.24	280.86	285.18	25.02	52.23	−18.03	−15.13
DMU8	48.94	53.87	345.38	343.53	18.85	19.90	374.93	376.33	15.24	19.63	31.51	42.48
DMU9	126.12	163.94	588.52	619.87	29.09	34.10	659.37	688.77	44.54	41.14	208.37	212.11
DMU10	2,204.59	2,252.80	21,456.07	19,662.15	844.84	829.44	22,758.58	20,878.59	464.34	404.14	−4,920.77	−4,596.52
DMU11	225.65	227.30	517.56	544.43	46.76	57.28	511.09	501.17	19.19	12.00	406.74	411.64
DMU12	277.35	268.43	1,127.42	1,135.68	67.35	68.06	1,193.96	1,192.60	8.98	6.05	86.35	85.17
DMU13	176.21	210.65	1,922.96	2,072.90	66.55	67.58	2,016.90	2,164.38	40.14	37.07	266.55	295.56
DMU14	110.18	107.74	1,362.00	1,290.00	319.10	311.22	1,743.03	1,644.14	82.26	67.53	1,106.67	1,105.78
DMU15	187.13	168.09	317.18	309.49	22.88	22.12	344.28	335.18	4.79	4.10	−145.64	−173.85
DMU16	157.67	150.90	1.844.77	1.813.44	137.11	132.31	2,097.38	2,066.96	121.79	130.88	549.79	1,023.06
DMU17	186.10	221.06	403.77	499.94	27.88	29.14	480.62	585.03	24.80	28.73	51.79	56.00
DMU18	100.20	107.81	784.66	988.72	47.44	48.82	837.89	1,044.86	0.09	0.09	91.96	91.81
DMU19	335.52	337.99	6,278.05	6,271.69	267.56	266.78	6,730.52	6,721.42	255.74	330.62	−1,942.50	−1,812.81
DMU20	28.86	28.70	331.11	333.48	14.41	14.62	352.01	356.74	5.78	8.41	11.35	11.16

Source: calculated by researchers.

**Table 5:** Average MAPE of DMUs.

DMUs	Average MAPE
DMU1	1.626%
DMU2	5.663%
DMU3	2.256%
DMU4	4.001%
DMU5	5.021%
DMU6	0.790%
DMU7	9.307%
DMU8	2.180%
DMU9	9.525%
DMU10	2.459%
DMU11	1.369%
DMU12	7.792%
DMU13	2.069%
DMU14	1.124%
DMU15	14.513%
DMU16	7.046%
DMU17	2.089%
DMU18	15.664%
DMU19	5.527%
DMU20	16.422%`
Average of all MAPEs	**5.822%**

**Table 6:** Pearson correlation coefficient.

Correlation coefficient	Degree of correlation
>0.8	Very high
0.6–0.8	High
0.4–0.6	Medium
0.2–0.4	Low
<0.2	Very low

**Table 7:** Correlation of input and output data in 2009.

	Fixed assets	Cost of goods sold	Operating expenses	Revenues	Operating income	Retained earning
Fixed assets	1	0.9918877	0.9948622	0.9934644	0.9870813	0.5567119
Cost of goods sold	0.9918877	1	0.9883356	0.9998524	0.9707767	0.4694186
Operating expenses	0.9948622	0.9883356	1	0.9906894	0.9908935	0.5716045
Revenues	0.9934644	0.9998524	0.9906894	1	0.974622	0.4824481
Operating income	0.9870813	0.9707767	0.9908935	0.974622	1	0.6383985
Retained earning	0.5567119	0.4694186	0.5716045	0.4824481	0.6383985	1

**Table 8:** Correlation of input and output data in 2010.

	Fixed assets	Cost of goods sold	Operating expenses	Revenues	Operating income	Retained earning
Fixed assets	1	0.9961601	0.9946756	0.9966511	0.9969196	0.6693217
Cost of goods sold	0.9961601	1	0.9897864	0.9999604	0.9965469	0.6249093
Operating expenses	0.9946756	0.9897864	1	0.9910043	0.995016	0.7097266
Revenues	0.9966511	0.9999604	0.9910043	1	0.9970943	0.6303295
Operating income	0.9969196	0.9965469	0.995016	0.9970943	1	0.6591707
Retained earning	0.6693217	0.6249093	0.7097266	0.6303295	0.6591707	1

**Table 9:** Correlation of input and output data in 2011.

	Fixed assets	Cost of goods sold	Operating expenses	Revenues	Operating income	Retained earning
Fixed assets	1	0.9950823	0.9971701	0.9956782	0.9932562	0.7714851
Cost of goods sold	0.9950823	1	0.9916572	0.99997	0.9941673	0.7180692
Operating expenses	0.9971701	0.9916572	1	0.9925723	0.9901189	0.7850506
Revenues	0.9956782	0.99997	0.9925723	1	0.9946349	0.7221627
Operating income	0.9932562	0.9941673	0.9901189	0.9946349	1	0.7310947
Retained earning	0.7714851	0.7180692	0.7850506	0.7221627	0.7310947	1

**Table 10:** Correlation of input and output data in 2012.

	Fixed assets	Cost of goods sold	Operating expenses	Revenues	Operating income	Retained earning
Fixed assets	1	0.999063	0.9965849	0.9991703	0.9979143	0.8264087
Cost of goods sold	0.999063	1	0.9956829	0.9999824	0.9978927	0.8122929
Operating expenses	0.9965849	0.9956829	1	0.9961923	0.9957381	0.8506753
Revenues	0.9991703	0.9999824	0.9961923	1	0.9981097	0.8150393
Operating income	0.9979143	0.9978927	0.9957381	0.9981097	1	0.8284635
Retained earning	0.8264087	0.8122929	0.8506753	0.8150393	0.8284635	1

**Table 11:** Efficiency and ranking before strategic alliances.

Rank	DMUs	Scores
1	DMU14	3.9649155
2	DMU11	1.6592766
3	DMU7	1.6289051
4	DMU9	1.5433191
5	DMU16	1.3485030
6	DMU2	1.3480462
7	DMU20	1.2457183
8	DMU19	1.2182798
9	DMU5	1.2120785
10	DMU4	1.2055717
11	DMU6	1.1491487
12	DMU10	1.1317559
13	DMU8	1.0661248
14	DMU17	1.0583728
15	DMU1	1
16	DMU13	0.9527483
17	DMU3	0.8578195
18	DMU18	0.7987767
19	DMU15	0.7192282
20	DMU12	0.6572566

**Table 12:** Efficiency and ranking after strategic alliances.

Rank	DMUs	Scores
1	DMU14	3.811933878
2	DMU11	1.659276566
3	DMU7	1.628905128
4	DMU9	1.543319054
5	DMU16	1.348503005
6	DMU2	1.287551129
7	DMU20	1.245718314
8	DMU19	1.218279755
9	DMU5	1.212078483
10	DMU4	1.205571702
11	DMU6	1.149148703
12	DMU10	1.119700896
13	DMU8	1.066124773
14	DMU17	1.058372798
15	DMU1 + DMU19	1.004835943
16	DMU1 + DMU5	1.00244846
17	DMU1 + DMU6	1.001653634
18	DMU1 + DMU4	1.000788967
19	DMU1 + DMU14	1
19	DMU1 + DMU10	1
19	DMU1 + DMU2	1
22	DMU1 + DMU16	0.999519376
23	DMU1 + DMU13	0.997682383
24	DMU1 + DMU9	0.996586955
25	DMU1	0.99637024
26	DMU1 + DMU20	0.996194591
27	DMU1 + DMU8	0.996148939
28	DMU1 + DMU18	0.995511442
29	DMU1 + DMU7	0.995424094
30	DMU1 + DMU17	0.994474568
31	DMU1 + DMU3	0.99402304
32	DMU1 + DMU11	0.993393149
33	DMU1 + DMU15	0.991332723
34	DMU1 + DMU12	0.991328063
35	DMU13	0.952748304
36	DMU3	0.857819532
37	DMU18	0.79877668
38	DMU15	0.719228198
39	DMU12	0.65725664

**Table 13:** The good alliance partnership.

Virtual alliance	Target DMU1 ranking (1)	Virtual alliance ranking (2)	Difference: (1) − (2)
DMU1 + DMU19	25	15	10
DMU1 + DMU5	25	16	9
DMU1 + DMU6	25	17	8
DMU1 + DMU4	25	18	7
DMU1 + DMU14	25	19	6
DMU1 + DMU10	25	19	6
DMU1 + DMU2	25	19	6
DMU1 + DMU16	25	22	3
DMU1 + DMU13	25	23	2
DMU1 + DMU9	25	24	1

**Table 14:** The unqualified alliance partnership.

Virtual alliance	Target DMU1 ranking (1)	Virtual alliance ranking (2)	Difference: (1) − (2)
DMU1 + DMU20	25	26	−1
DMU1 + DMU8	25	27	−2
DMU1 + DMU18	25	28	−3
DMU1 + DMU7	25	29	−4
DMU1 + DMU17	25	30	−5
DMU1 + DMU3	25	31	−6
DMU1 + DMU11	25	32	−7
DMU1 + DMU15	25	33	−8
DMU1 + DMU12	25	34	−9

**Table 15:** The impossible alliance partners.

DMUs	Rank before alliance	Rank after alliance
DMU19	8	15
DMU5	9	16
DMU6	11	17
DMU4	10	18
DMU14	1	19
DMU10	12	19
DMU2	6	19
DMU16	5	22
DMU9	4	24

## Sources from Websites

*Bloomberg Business Week*, available at http://www.businessweek.com/;* Bloomberg news*, available at http://www.bloomberg.com/; Chartered Institute of Management Accountants, “*Global Manufacturing report,*” “August 2010”, available at http://www.cimaglobal.com/; Saitech Company's website: http://www.saitech-inc.com/index.asp.
